# Can Plasma ADAMTS13 Differentiate Patients With Pulmonary Arterial Hypertension From Other Forms of Pulmonary Hypertension and Dyspnea Control Patients

**DOI:** 10.1016/j.chpulm.2025.100178

**Published:** 2025-05-19

**Authors:** Kriss Kania, Abdulla Ahmed, Salaheldin Ahmed, Adam Engel Sällberg, Karin Tran-Lundmark, Jørn Carlsen, Göran Rådegran

**Affiliations:** a Department of Clinical Sciences Lund, Cardiology, Lund University, Lund, Sweden; b The Hemodynamic Lab, Section for Heart Failure and Valvular Disease, VO, Heart and Lung Medicine, Skåne University Hospital, Lund, Sweden; c Department of Education and Research, Helsingborg Hospital, Helsingborg, Sweden; d Department of Experimental Medical Science, Lund University, Lund, Sweden; e The Pediatric Heart Center, Skåne University Hospital, Lund, Sweden; f Wallenberg Center for Molecular Medicine, Lund University, Lund, Sweden; g Department of Cardiology, Copenhagen University Hospital, Rigshospitalet, Copenhagen, Denmark; h Department of Clinical Medicine, Faculty of Health and Medical Sciences, University of Copenhagen, Copenhagen, Denmark

**Keywords:** ADAM-TS13, ADAMTS-13, diagnosis, discrimination, HFpEF, HFrEF, thrombosis

## Abstract

**Background:**

Multimarker panels of blood-borne biomarkers could aid in shortening the diagnostic delay of pulmonary arterial hypertension (PAH).

**Research Question:**

Can any of the 61 proteins included in the study, related to pathways known to be involved in PAH pathophysiology (eg, coagulation, inflammation, fibrosis), differentiate PAH from other forms of pulmonary hypertension (PH) and/or dyspnea and thus aid in diagnosing PAH?

**Study Design and Methods:**

Plasma samples were collected from 55 healthy control participants and 355 patients at diagnosis, including PAH (n = 95), chronic thromboembolic PH (n = 54), heart failure with preserved ejection fraction with PH (n = 58), heart failure with reduced ejection fraction with PH (n = 64), a non-PH dyspnea control group with heart failure (n = 45), and an independent external PAH cohort (n = 39) used as external validation of protein levels in the PAH group of the discovery cohort. Plasma levels of the 61 proteins were analyzed using proximity extension assay.

**Results:**

ADAMTS13 plasma levels differed in patients with PAH compared with the 3 other PH groups and the non-PH dyspnea group (*P* < .05). In univariable (OR, 0.90; 95% CI, 0.84-0.96) and multivariable logistic regression models adjusted for age and sex (OR, 0.89; 95% CI, 0.83-0.96), ADAMTS13 was additionally able to differentiate PAH from the other combined disease groups. In the external validation cohort, plasma ADAMTS13 levels in PAH were statistically equivalent to the discovery cohort (*P <* .002).

**Interpretation:**

Plasma ADAMTS13 may be a diagnostic biomarker in PAH with the ability to differentiate patients with PAH from other dyspnea groups and is an interesting protein for inclusion in future studies of multimarker panels. Further validation in larger cohorts is warranted.

**Take-Home Points**

**Study Question:** Do any of the 61 proteins investigated in this study possess the ability to differentiate patients with pulmonary arterial hypertension (PAH) from patients with dyspnea from other causes, including other pulmonary hypertension groups?

**Results:** Plasma levels of ADAMTS13 were lower in pulmonary arterial hypertension than the other disease groups (*P* < .05), and were able to differentiate between these groups in a multivariable logistic regression adjusted for age and sex, with an OR of 0.89.

**Interpretation:** Plasma ADAMTS13 may be a diagnostic biomarker in pulmonary arterial hypertension, and is an interesting protein for inclusion in future studies of multimarker panels in PAH diagnostics.

Pulmonary hypertension (PH) is classified into 5 distinct groups related to their specific etiologies and pathophysiological mechanisms.^1^ Pulmonary arterial hypertension (PAH) (World Health Organization group I PH) specifically is a rare disease characterized by an increased pulmonary vascular resistance (PVR) related to multifactorial pathologic processes involving increased deposition of extracellular matrix and intimal fibrosis, vasoconstriction, microthrombosis, inflammation, and dysregulated smooth muscle cell and endothelial cell proliferation.^2^^,^^3^ This progressive pulmonary vasculopathy leads to right-sided heart failure (HF) and premature death.^1^ Over the past 3 decades, an increased pathophysiological understanding of the dysfunctional signaling systems in the pulmonary vasculature has led to the identification of targets for PAH-specific therapy, leading to improved long-term outcomes.^4^, ^5^, ^6^

Despite the improvement of treatment strategies and outcomes, early identification and confirmation of a PAH diagnosis is still challenging due to unspecific symptoms, and the mean diagnostic delay of 24 to 30 months has remained principally unchanged since 1987.^7^^,^^8^ One of the most important points highlighted in clinical guidelines in the diagnosis of unclear dyspnea is early suspicion of the occurrence of PH and fast track referrals to expert centers for patients with a high likelihood of severe forms (eg, PAH).^1^ Consequently, new diagnostic approaches that could aid in differentiating the cause of dyspnea and facilitate an earlier diagnosis of PAH are called for.^1^

Blood-borne biomarkers associated with PAH could provide a possible modality to achieve earlier diagnosis, and substantial efforts have been made to study proteins that could aid in PH diagnosis, differentiation, and prognosis.^1^^,^^9^, ^10^, ^11^, ^12^ We have previously demonstrated plasma ADAMTS13 to be a potential biomarker differentiating PAH from healthy control patients, and other related PH groups and a HF without pulmonary hypertension (HF-Non-PH) dyspnea group.^13^ To validate these results in the present study, we have expanded our biobank pool of patients with PH and healthy control patients, and included an independent external validation cohort of patients with PAH. The examined set of interrelated proteins was also broadened compared with our previous study.

The aim of this study was to examine the plasma levels of a set of proteins included in pathways related to PAH pathophysiology (eg, coagulation, inflammation, fibrosis) for their diagnostic ability in differentiating between patients with PAH and other causes of dyspnea, with or without PH, and healthy control participants. The study also specifically aimed to verify plasma ADAMTS13 as a potential biomarker for this purpose.

## Materials and Methods

### Study Population and Blood Samples

The discovery cohort included 55 self-reported healthy control participants with no history of cardiovascular disease, and 316 patients diagnosed at Skåne’s University Hospital in Lund, Sweden. Of these 316 patients, 45 had HF-Non-PH (HF-Non-PH dyspnea control patients) and 271 had PH, including 58 with heart failure with preserved ejection fraction and pulmonary hypertension (HFpEF-PH), 64 with heart failure with reduced ejection fraction and pulmonary hypertension (HFrEF-PH), 95 with PAH, and 54 with chronic thromboembolic pulmonary hypertension (CTEPH). Additionally, 39 patients with PAH diagnosed at Rigshospitalet in Copenhagen, Denmark, were included as an independent external PAH validation cohort. All participants were ≥ 18 years of age and provided written informed consent. The study was conducted in accordance with the ethical standards defined in the declarations of Helsinki and Istanbul, and was approved by the ethical boards in Lund, Sweden (Dnr: 2010/114, 2010/442, 2011/368, 2011/777, 2015/270), Region H (H-17021130) and Datatilsynet, Copenhagen, Denmark (J.nr. 2022-522-0452).

Data for this study were obtained from venous blood samples collected between October 2011 and December 2021 and stored at −80 °C in Lund Cardio Pulmonary Registry, a cohort of Region Skåne’s biobank, and between December 2018 and August 2022 for the external Copenhagen cohort from the Danish Pulmonary Hypertension (DAN-PH) registry. Nonfasting venous blood samples were collected from all study participants. Patient samples in the discovery and validation cohorts were collected during or immediately preceding the diagnostic right heart catheterization (RHC). The patients’ listed medications are a baseline of their current medication intake at diagnosis and are therefore unrelated to their PH diagnosis.

### Plasma Protein Analysis

Proximity extension assay (PEA) was used to analyze 61 proteins related to coagulation, inflammation, fibrosis, and other pathways related to PH. Proseek Multiplex immunoassay kits (Cardiovascular disease II and III and Oncology III panels; Olink Proteomics) were used. In PEA, oligonucleotide-labeled antibodies are mixed with the sample. The antibodies bind pairwise to the protein target and create a target sequence for a polymerase chain reaction. This sequence is then detected and quantified using quantitative real-time polymerase chain reaction. To account for interplate variations, protein level normalization, and data quality control, internal controls were added to each sample, and external controls were added as separate samples. Protein levels obtained using PEA are expressed as arbitrary units on a linear, normalized protein expression scale.^14^ The list of all the analyzed proteins and their abbreviations can be found in e-Table 1.

### Hemodynamics

Hemodynamic data for the patients were acquired during RHC as part of routine clinical examinations, using a Swan Ganz catheter (Baxter Health Care Corp), predominantly inserted via the right internal jugular vein in a supine position. PH was diagnosed and divided into the various subgroups, according to prevailing European Society of Cardiology/European Respiratory Society guidelines.^15^^,^^16^ HFpEF-PH and HFrEF-PH were defined according to prevailing guidelines at the time of diagnosis as HF with an ejection fraction ≥ 50% and < 50%, respectively, combined with a mean pulmonary arterial pressure ≥ 25 mmHg and pulmonary arterial wedge pressure (PAWP) ≥ 15 mmHg.^17^^,^^18^

### Statistics

Continuous descriptive variables are presented as medians with interquartile ranges (IQRs), unless otherwise stated. Data were tested for normal distribution visually using histograms. The Mann-Whitney *U* test and Kruskal-Wallis test by ranks with subsequent post hoc analysis using the uncorrected Dunn test, receiver operating characteristic (ROC) curves, and multiple logistic regression models were used as appropriate. DeLong test for comparing correlated ROC curves was used to evaluate and compare the performance of the regression models.^19^ Equivalence testing using a Wilcoxon-based two 1-sided test (TOST) method for unpaired samples was performed to validate the levels of relevant proteins in the discovery PAH cohort against the validation PAH cohort to strengthen the results of the study. Because the included proteins are not currently used clinically to differentiate between the present groups, choosing equivalency bounds becomes arbitrary. We chose IQR as the lower and upper bounds a priori to the TOST equivalence analysis, which we think is fairly conservative. The false discovery rate (FDR) was calculated using the 2-stage step-up method of Benjamini, Krieger, and Yekutieli and was used to account for mass significance. The Q values were chosen as the highest of 1%, 5%, or 10% while still maintaining a *P* value cutoff < .05. In analyses where the FDR was not applied, *P* < .05 was deemed statistically significant. The DeLong and TOST analyses were performed using R (R Foundation for Statistical Computing) with the pROC and TOSTER packages, respectively.^20^, ^21^, ^22^ All remaining analyses were performed using GraphPad Prism version 9.5.1 (GraphPad Software).

## Results

### Demographics

The population characteristics, including hemodynamics, comorbidities, and medications, can be found in Tables 1 and 2. Descriptive statistics including median levels and IQRs of each protein in the study population can be found in e-Table 2. The 95 patients with PAH in the Lund discovery cohort included 49 patients with idiopathic pulmonary arterial hypertension (IPAH), 40 with pulmonary arterial hypertension associated with connective tissue disease (CTD-APAH), and 6 with other PAH subtypes. In total, 7 were acute vasoresponders. Thirty-six of the 40 patients with CTD-APAH had systemic sclerosis-associated PAH. Of the 39 patients with PAH in the Copenhagen validation cohort, 20 had IPAH and 19 had CTD-APAH.

**Table 1:** Demographics of the Lund Discovery Cohort

Demographic	Control Participants (n = 55)		CTEPH (n = 54)		PAH (n = 95)		HFpEF-PH (n = 58)		HFrEF-PH (n = 64)		Non-PH HF (n = 45)	
	No. (%)	Median (IQR)	No. (%)	Median (IQR)	No. (%)	Median (IQR)	No. (%)	Median (IQR)	No. (%)	Median (IQR)	No. (%)	Median (IQR)
Female	29 (53)	NA	31 (57)	NA	78 (82)	NA	39 (67)	NA	11 (17)	NA	22 (49)	NA
Age, y	55	41 (26-51)	54	73 (66-78)	95	70 (57-76)	58 (100)	76 (71-82)	64	53 (43-58)	45	62 (57-70)
Height, cm	55	173 (167-180)	54	169 (164-177)	94	163 (158-170)	58 (100)	167 (162-174)	62	180 (174-183)	45	174 (167-180)
Weight. kg	54	73 (62-81)	54	78 (71-86)	94	69 (60-78)	58 (100)	81 (74-98)	62	82 (72-91)	45	79 (68-94)
BSA, m^2^	54	1.87 (1.72-2.00)	54	1.89 (1.78-2.02)	94	1.75 (1.61-1.90)	58 (100)	1.91 (1.78-2.12)	62	2.01 (1.90-2.15)	44	2.00 (1.73-2.11)
Sao_2_, %	55	98 (97-99)	54	91 (87-94)	95	90 (86-95)	58 (100)	95 (91-97)	60	95 (93-97)	45	96 (94-97)
mAP, mm Hg	55	95 (90-102)	54	103 (94-114)	95	100 (89-107)	58 (100)	100 (92-108)	62	78 (75-84)	45	90 (81-98)
Creatinine, μmol/L	NA	NA	30	91 (73-113)	67	83 (70-112)	51 (88)	99 (82-131)	47	120 (94-135)	26	91 (73-110)
BNP, AU	NA	NA	54	32 (6-78)	95	41 (11-97)	58 (100)	28 (16-49)	64	143 (64-186)	45	28 (5-75)
Hypertension	NA	NA	21 (39)	NA	32 (34)	NA	32 (55)	NA	7 (11)	NA	8 (18)	NA
Diabetes mellitus	NA	NA	2 (4)	NA	14 (15)	NA	16 (28)	NA	4 (6)	NA	3 (7)	NA
Atrial fibrillation	NA	NA	6 (11)	NA	4 (4)	NA	36 (62)	NA	15 (23)	NA	9 (20)	NA
Stroke	NA	NA	1 (2)	NA	2 (2)	NA	6 (10)	NA	3 (5)	NA	3 (7)	NA
IHD	NA	NA	5 (9)	NA	11 (12)	NA	10 (17)	NA	5 (8)	NA	7 (16)	NA
Thyroid disease	3 (5)	NA	2 (4)	NA	16 (17)	NA	4 (7)	NA	3 (5)	NA	4 (9)	NA
mPAP, mm Hg	NA	NA	54 (100)	43 (37-49)	95 (100)	47 (38-55)	58 (100)	34 (30-43)	62 (97)	35 (29-40)	45 (100)	17 (14-19)
PAWP, mm Hg	NA	NA	54 (100)	10 (7-12)	95 (100)	8 (6-10)^a^	58 (100)	18 (17-22)	61 (95)	25 (20-29)	45 (100)	9 (6-14)
mRAP, mm Hg	NA	NA	54 (100)	7 (5-10)	95 (100)	7 (4-11)	58 (100)	11 (8-14)	62 (97)	13 (9-17)	45 (100)	4 (2-7)
RVSP, mm Hg	NA	NA	54 (100)	76 (63-88)	94 (99)	78 (64-88)	58 (100)	58 (46-71)	61 (95)	46 (39-55)	44 (98)	29 (24-33)
RVDP, mm Hg	NA	NA	54 (100)	1 (-1-4)	94 (99)	2 (-2-5)	58 (100)	3 (0-5)	61 (95)	6 (4-9)	44 (98)	1 (−1 to 4)
CO, L/min	NA	NA	54 (100)	4.0 (3.5-4.9)	95 (100)	3.9 (3.1-4.9)	58 (100)	4.4 (3.7-5.6)	61 (95)	3.4 (2.9-4.1)	44 (98)	4.4 (3.5-5.3)
PVR, WU	NA	NA	54 (100)	9.1 (6.1-11)	95 (100)	9.8 (6.9-13.6)	58 (100)	3.5 (2.5-4.3)	61 (95)	2.7 (2-3.7)	45 (100)	1.6 (1.1-1.9)
Calcium antagonists	NA	NA	0 (0)	NA	14 (15)	NA	0 (0)	NA	0 (0)	NA	0 (0)	NA
ACEi	NA	NA	3 (6)	NA	11 (12)	NA	14 (24)	NA	19 (30)	NA	5 (11)	NA
ARB	NA	NA	6 (11)	NA	4 (4)	NA	15 (26)	NA	13 (20)	NA	7 (16)	NA
ARNI	NA	NA	2 (4)	NA	0 (0)	NA	0 (0)	NA	1 (2)	NA	5 (11)	NA
Beta-blocker	NA	NA	9 (17)	NA	17 (18)	NA	34 (59)	NA	39 (61)	NA	19 (42)	NA
Loop diuretic	NA	NA	7 (13)	NA	12 (13)	NA	22 (38)	NA	37 (58)	NA	20 (44)	NA
MRA	NA	NA	3 (6)	NA	12 (13)	NA	9 (16)	NA	28 (44)	NA	19 (42)	NA
Cortisone/prednisolone	NA	NA	1 (2)	NA	3 (3)	NA	5 (9)	NA	2 (3)	NA	2 (4)	NA

Data are presented as median (interquartile range) or No. (%). ACEi = angiotensin-converting enzyme inhibitor; ARB = angiotensin receptor blocker; ARNI = angiotensin receptor-neprilysin inhibitor; AU = arbitrary unit; BNP = brain natriuretic peptide; BSA = body surface area; CO = cardiac output; CTEPH = chronic thromboembolic pulmonary hypertension; HFpEF-PH = heart failure with preserved ejection fraction and pulmonary hypertension; HFrEF-PH = heart failure with reduced ejection fraction and pulmonary hypertension; IHD = ischemic heart disease; IQR = interquartile range; mAP = mean arterial pressure; mPAP = mean pulmonary arterial pressure; MRA = aldosterone receptor antagonist; mRAP = mean right atrial pressure; Non-PH HF = heart failure without pulmonary hypertension; PAH = pulmonary arterial hypertension; PAWP = pulmonary arterial wedge pressure; PVR = pulmonary vascular resistance; RVDP = right ventricular diastolic pressure; RVSP = right ventricular systolic pressure; Sao_2_ = arterial oxygen saturation; WU = Wood unit.

a Ten of 95 patients with PAH had a PAWP between 13 and 15. Of the 49 patients with IPAH in the PAH group, 7 had a PAWP between 13 and 15.

**Table 2:** Demographics of the Copenhagen Validation Cohort

Demographic	Copenhagen PAH (n = 39)	
	No. (%)	Median (IQR)
ADAMTS13, AU	39 (100)	25.7 (23.6-28.2)
Female	30 (77)	NA
Age, y	39 (100)	67 (54-76)
BSA, m^2^	39 (100)	1.9 (1.7-2.0)
6MWT, m	37 (95)	317 (223-378)
mAP, mm Hg	39 (100)	95 (91-106)
BNP, AU	39 (100)	45 (13-136)
Creatinine, μmol/L	39 (100)	84 (69-97)
mPAP, mm Hg	39 (100)	47 (34-59)
PAWP, mm Hg	39 (100)	11 (9-14)
mRAP, mm Hg	39 (100)	9 (8-12)
CO, L/min	39 (100)	5.1 (3.8-6)
PVR, WU	39 (100)	6.6 (4.6-9.6)
Hypertension	9 (23)	NA
Diabetes mellitus	3 (8)	NA
Atrial fibrillation	0 (0)	NA
Stroke	1 (3)	NA
IHD	1 (3)	NA
Thyroid disease	0 (0)	NA

6MWT = 6-minute walking test; AU = arbitrary units; BNP = brain natriuretic peptide; BSA = body surface area; CO = cardiac output; IHD = ischemic heart disease; IQR = interquartile range; mAP = mean arterial pressure; mPAP = mean pulmonary arterial pressure; mRAP = mean right atrial pressure; PAH = pulmonary arterial hypertension; PAWP = pulmonary arterial wedge pressure; PVR = pulmonary vascular resistance; WU = Wood unit.

### Plasma ADAMTS13, Glyoxalase I, and Protein-Glutamine Gamma-Glutamyltransferase 2 Differed Significantly in Patients With PAH Compared With Other Patient Groups

**Figure 1:**
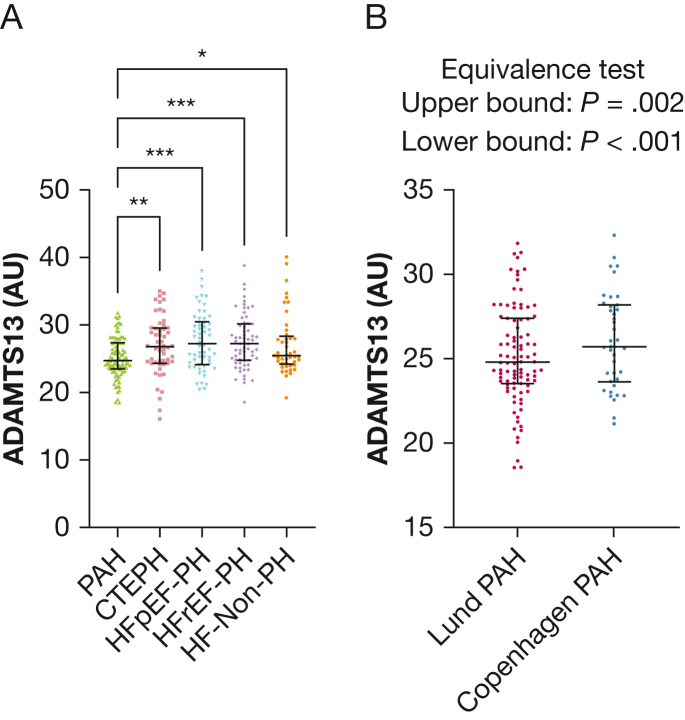
A, Levels of ADAMTS13 in the 5 patient groups and (B) equivalence test between the Lund PAH group and the Copenhagen external validation PAH group. An outlier of 65.41 AU in the Lund ADAMTS13 PAH group was removed from the graph for improved legibility. ADAMTS13 indicates a disintegrin and metalloproteinase with thrombospondin motifs 13. ∗*P* < .05. ∗∗*P* < .01. ∗∗∗*P* < .001. AU = arbitrary unit; CTEPH = chronic thromboembolic pulmonary hypertension; HFpEF-PH = pulmonary hypertension associated with heart failure with preserved ejection fraction; HFrEF-PH = pulmonary hypertension associated with heart failure with reduced ejection fraction; HF-Non-PH = heart failure without pulmonary hypertension; PAH = pulmonary arterial hypertension.

**Figure 2:**
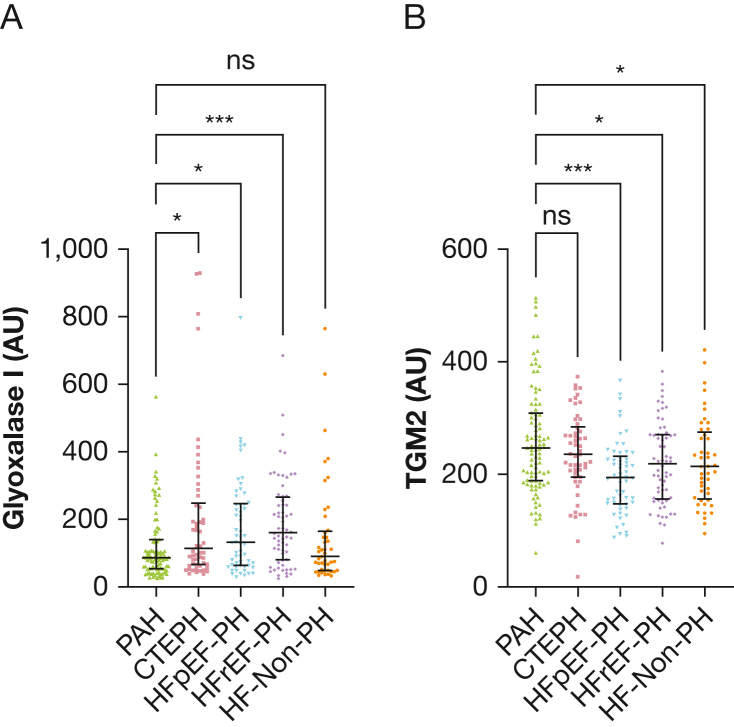
A, Levels of glyoxalase I and (B) TGM2 in the 5 patient groups. ∗*P* <0.05. ∗∗∗*P* < .001. AU = arbitrary unit; CTEPH = chronic thromboembolic pulmonary hypertension; HFpEF-PH = pulmonary hypertension associated with heart failure with preserved ejection fraction; HFrEF-PH = pulmonary hypertension associated with heart failure with reduced ejection fraction; HF-Non-PH = heart failure without pulmonary hypertension; ns = not significant; PAH = pulmonary arterial hypertension; TGM2 = protein-glutamine gamma-glutamyltransferase 2.

After Kruskal-Wallis and FDR analysis of the 61 proteins, a total of 22 proteins differed in plasma levels between PAH and at least 1 of the other patient groups (*P* < .005; FDR, < 1%) (e-Table 3). Next, plasma levels of these 22 proteins were compared between the PAH group and the CTPEH, HFpEF-PH, HFrEF-PH, and HF-Non-PH groups. Plasma ADAMTS13, glyoxalase I, and protein-glutamine gamma-glutamyltransferase 2 (TGM2) were the only proteins with PAH levels that differed compared with at least 3 of the 4 other patient groups. Specifically, ADAMTS13 plasma levels in PAH were lower than all 4 other respective groups (*P* < .05) (Fig 1). Glyoxalase I plasma levels were lower in PAH compared with CTEPH, HFpEF-PH, and HFrEF-PH (*P* < .05), whereas TGM2 plasma levels were higher in PAH compared with HFpEF-PH, HFrEF-PH, and HF-Non-PH (*P* < .05) (e-Table 4, Fig 2).

### ADAMTS13 Differentiates PAH From Other Dyspnea-Related Groups

Plasma ADAMTS13, glyoxalase I, and TGM2 were further analyzed for their differentiating ability using logistic regressions and ROC curves (Table 3). Univariable logistic regressions showed decreasing odds of having PAH with an increase in plasma levels of ADAMTS13 (OR, 0.90; 95% CI, 0.84-0.96) and glyoxalase I (OR, 0.99; 95% CI, 0.99-1.00), whereas increasing TGM2 levels yielded increasing odds (OR, 1.01; 95% CI, 1.00-1.01). All 3 proteins also had significant ROC curves (*P* < .05) with ADAMTS13 having the highest area under the curve (AUC) (OR, 0.65; 95% CI, 0.58-0.71).

**Table 3:** Univariable and Multivariable Logistic Regression Models of ADAMTS13, Glyoxalase I, and TGM2

Proteins and Covariables	OR (95% CI)	*P* Value	AUC (95% CI)
ADAMTS13, AU	0.90 (0.84-0.96)	.002^a^	0.65 (0.58-0.71)
Glyoxalase I, AU	0.99 (0.99-1.00)	.001^a^	0.62 (0.55-0.68)
TGM2, AU	1.01 (1.00-1.01)	.001^a^	0.62 (0.55-0.69)
Age, y	1.01 (0.99-1.02)	.572	0.53 (0.46-0.60)
Sex, female	5.26 (2.98-9.72)	< .001^a^	0.68 (0.62-0.74)
Multivariable logistic regression models and ROC analyses			
Model 1			0.76 (0.70-0.82)
ADAMTS13, AU	0.89 (0.83-0.96)	.002^a^	
Age, y	0.99 (0.97-1.01)	.260	
Sex, female	5.78 (3.18-11.00)	< .001^a^	
Model 2			0.76 (0.70-0.81)
ADAMTS13, AU	0.93 (0.85-1.00)	.079	
Glyoxalase I, AU	1.00 (0.99-1.00)	.086	
Age, y	0.99 (0.97-1.00)	.339	
Sex, female	5.50 (3.03-10.49)	< .001^a^	
Model 3			0.77 (0.72-0.83)
ADAMTS13, AU	0.91 (0.84-0.98)	.011^a^	
TGM2, AU	1.01 (1.00-1.01)	.002^a^	
Age, y	0.99 (0.97-1.01)	.330	
Sex, female	5.61 (3.07-10.78)	< .001^a^	
Model 4			0.77 (0.72-0.83)
ADAMTS13, AU	0.96 (0.88-1.05)	.411	
Glyoxalase I, AU	1.00 (0.99-1.00)	.029^a^	
TGM2, AU	1.01 (1.00-1.01)	< .001^a^	
Age, y	0.99 (0.98-1.01)	.504	
Sex, female	5.24 (2.85-10.09)	< .001^a^	
Comparisons of AUC between models			
Model 1 vs model 2		.531	
Model 1 vs model 3		.413	
Model 1 vs model 4		.432	

Multivariable models also include age and sex. Proteins that showed an ability to differentiate PAH from at least 3 of the 4 other disease groups qualified for subsequent differentiation analysis using ROC curves and multiple logistic regression models. Both ROC curves and regression models compared the PAH group against all 4 remaining disease groups combined. AU, arbitrary unit; AUC, area under the receiver operating characteristic curve; PAH = pulmonary arterial hypertension; ROC = receiver operating characteristic; TGM2, protein-glutamine gamma-glutamyltransferase 2.

a *P* < .05.

To further assess the differentiating ability of the proteins, 4 multivariable logistic regression models were fitted with a subsequent ROC analysis (Table 3). Model 1 included ADAMTS13, age, and sex; model 2 included the variables in model 1 with the addition of glyoxalase I; model 3 replaced glyoxalase I with TGM2; and model 4 included ADAMTS13, glyoxalase I, TGM2, age, and sex. All 4 models showed similar AUCs, and a subsequent DeLong test showed that the models including TGM2 and glyoxalase I did not significantly differ in terms of AUC from the model that only included ADAMTS13. The interpretation of the AUC in the models should be done with caution because it is impacted by female individuals being heavily overrepresented in the PAH group, thus making sex a clear differentiator of PAH as seen in the univariable logistic regressions. Nonetheless, plasma ADAMTS13 remained a significant differentiator of PAH in the first model independently of age and sex (OR, 0.89; 95% CI, 0.83-0.96).

To validate these results, equivalence testing of plasma ADAMTS13 levels between the Lund discovery PAH cohort and validation Copenhagen PAH cohort was performed. In the Wilcoxon-based TOST analysis, the groups were deemed to be statistically equivalent, with *P* = .002 and *P* < .001 for the upper and lower bounds, respectively (Fig 1).

Additionally, the plasma levels of all 61 proteins were compared between the patients with PAH and healthy control participants with subsequent FDR (e-Table 5). The plasma levels of 28 proteins differed significantly between the PAH group and healthy control participants (*P* < .008; FDR, < 1%), whereas ADAMTS13 plasma levels did not (*P* = .087). In a Mann-Whitney subgroup analysis, ADAMTS13 plasma levels in healthy control participants differed when compared with patients with CTD-APAH (*P* = .044), but not when compared with patients with IPAH (*P* = .296).

### ADAMTS13 Levels Correlated With Mean Pulmonary Arterial Pressure and PVR

Correlations were made using the Spearman rank coefficient (r_s_) between ADAMTS13 plasma levels in patients with PAH and several of their hemodynamic parameters measured during the RHC before treatment (Table 4). Plasma ADAMTS13 correlated weakly, albeit significantly, with mean pulmonary arterial pressure (r_s_ = 0.20; *P* < .05) and PVR (r_s_ = 0.22; *P* < .05).

**Table 4:** Correlations Between ADAMTS13 Baseline Levels Before Treatment in Patients With PAH in the Lund Discovery Cohort and Their Hemodynamic Parameters

Parameter	No.	r_s_	*P* Value
mPAP, mm Hg	95	0.20	.048^a^
PAWP, mm Hg	95	0.14	.172
mRAP, mm Hg	95	0.18	.091
SVI, mL/beat/m^2^	94	−0.09	.370
CI, L/min/m^2^	94	−0.18	.093
PVR, WU	95	0.22	.031^a^
RVSWI, mm Hg × mL/m^2^	94	0.03	.814
LVSWI, mm Hg × mL/m^2^	94	−0.05	.666
PAC, mL/mm Hg	95	−0.17	.108
NT-proBNP, ng/L	93	0.04	.712

CI = cardiac index; LVSWI = left ventricular stroke work index; mPAP = mean pulmonary arterial pressure; mRAP = mean right atrial pressure; NT-proBNP = N-terminal pro-brain natriuretic peptide; PAC = pulmonary arterial compliance; PAWP = pulmonary arterial wedge pressure; PVR = pulmonary vascular resistance; r_s_ = Spearman coefficient; RVSWI = right ventricular stroke work index; SVI = stroke volume index; WU = Wood units.

a *P* < .05.

## Discussion

Establishment and growth of new biobanks and novel, more efficient screening methods have stimulated the interest in using blood-borne biomarkers to decipher pathophysiological mechanisms and refine diagnostics and for prognostication and risk stratification.^23^^,^^24^ The complex pathophysiology of PAH involves numerous altered signaling systems, giving rise to research examining a wide range of proteins, including markers of inflammation, endothelial dysfunction, and coagulation.^1^^,^^23^^,^^24^ Despite these efforts, brain natriuretic peptides currently remain the only clinically used biomarkers in PAH.^1^ With this in mind, many reviews of the topic have argued it unlikely that any single marker could encompass all the necessary information about a particular patient, and that a likely way forward is the use of multimarker panels, combining several biomarkers that each bring unique information to improve diagnostic accuracy.^23^^,^^25^, ^26^, ^27^ Therefore, there is a need to map the plasma proteome to find novel candidate biomarkers that could be part of such a future biomarker panel. In this study, we found that plasma levels of ADAMTS13 were lower in patients with PAH compared with patients with CTEPH, HFrEF-PH, and HFpEF-PH as well as a HF-Non-PH dyspnea control group. In logistic regression and ROC analyses, ADAMTS13 was able to differentiate patients with PAH from the other groups and retained this ability when adjusted for age and sex in a multivariable regression analysis. Moreover, plasma ADAMTS13 levels in the PAH group were tested against an independent external validation PAH cohort and were deemed statistically equivalent. Although ADAMTS13 alone is unlikely to be sufficient for clinically differentiating patients with PAH, it could serve as a component of future multimarker panels.

ADAMTS13 is a protein in the a disintegrin-like and metalloproteinase with thrombospondin motifs family.^28^ It circulates in blood and is secreted as an active enzyme primarily from hepatic stellate cells; however, it has also been found to be expressed in endothelial cells and arterial and arteriolar vascular smooth muscle cells.^28^, ^29^, ^30^ In the circulation, it interacts with its only known substrate, von Willebrand factor (vWF) by proteolyzing large vWF multimers.^29^, ^30^, ^31^ Under conditions of high shear stress, vWF unfolds and thereby increases its platelet-binding activity leading to increased hemostasis. ADAMTS13 regulates this behavior by cleaving vWF multimers into smaller and less active fragments, thus inhibiting thrombosis in the circulation.^29^^,^^30^^,^^32^^,^^33^ ADAMTS13 itself is resistant to natural protease inhibitors in blood, and the mechanisms behind regulating its proteolytic activity in the circulation are thus unclear.^31^

Studies are however indicating that ADAMTS13 has functions outside of its antithrombotic properties (eg, regulating inflammation, angiogenesis, extracellular matrix degradation).^30^ The functions of ADAMTS13 make it a relevant and established protein in several diseases, most notably thrombotic thrombocytopenic purpura. In thrombotic thrombocytopenic purpura, ADAMTS13 activity or concentration is severely diminished, enabling large vWF multimers to form in parts of the cardiovascular system, leading to microangiopathy and systemic microvascular thrombosis.^29^^,^^32^ A dysregulated ADAMTS13-vWF axis with abnormal ADAMTS13 levels and activity has also been observed in other conditions involving vascular inflammation and thrombosis (eg, disseminated intravascular coagulation, sepsis, ischemic stroke, chronic HF, myocardial infarction).^30^^,^^33^^,^^34^ Lower ADAMTS13 levels have also been found in liver cirrhosis, decreasing with increased cirrhotic severity.^35^, ^36^, ^37^ To our knowledge, none of the population in the present study had these comorbidities at the time of blood sampling, except for HF.

Previous studies have also found ADAMTS13 levels to be slightly decreased in healthy people ≥ 65 years of age.^35^^,^^36^ Notably, the patient groups in the present study are of similar age, and ADAMTS13 remained significant after adjusting for age in the multivariable models. However, contrary to our previous study, ADAMTS13 levels in patients with PAH did not differ significantly from healthy control participants. We additionally conducted a multivariable linear regression analysis to assess if the levels differed between healthy control participants and patients with PAH when adjusted for age and sex, but PAH remained a statistically nonsignificant predictor of ADAMTS13 when compared with healthy control participants (*P* ≈ .78). This difference could partially be attributed to a different ratio of patients with CTD-APAH and patients with IPAH in the PAH group. In the previous study, 25 patients had CTD-APAH and 23 had IPAH/familial PAH, compared with 40 and 49 in this study, respectively, possibly skewing the results. Nonetheless, ADAMTS13 levels in PAH seem to remain similar to healthy control participants, but appear to be elevated in other dyspnea groups. Furthermore, although it is an interesting observation that ADAMTS13 levels do not differ in PAH compared with healthy control participants, it is not necessarily a clinically relevant finding. Due to its low prevalence, screening for PAH in a healthy, asymptomatic population is not feasible.^38^ In a clinical setting, PAH will need to be differentiated against symptomatic individuals with other pathologic conditions, such as the dyspnea group without PH included in the present study, rather than against healthy, asymptomatic control participants.

Despite the link between plasma ADAMTS13 levels and several pathologic cardiovascular conditions, the amount of previously published research studying plasma ADAMTS13 in PH is very limited. One previous study has found the ADAMTS13-vWF axis to be dysregulated in CTEPH, with patients with CTEPH having lower ADAMTS13 levels than healthy control participants.^39^ ADAMTS13 levels remained low in CTEPH after pulmonary endarterectomy, suggesting the lower ADAMTS13 levels could be related to the vascular remodeling process observed in CTEPH pathophysiology rather than being directly correlated to pulmonary pressures; however, notably, the present study did find a weak correlation between ADAMTS13 and mean pulmonary arterial pressure in PAH.^39^ Similar to the present study, ADAMTS13 levels did not differ between patients with IPAH and healthy control participants.^39^ To our knowledge, no other studies linking plasma ADAMTS13 levels to PH have been published, making our previous study the first to demonstrate this protein as a differentiator of PAH from other PH subtypes.^13^ In PAH, the local release of inflammatory cytokines and growth factors leads to vascular remodeling, notably involving proliferation of dysfunctional vascular smooth muscle cells and endothelial cells, both of which have been shown to express ADAMTS13 under healthy physiological conditions.^2^^,^^3^^,^^30^^,^^31^ In situ microthrombosis is another prominent feature of PAH.^2^^,^^3^ We therefore hypothesize that ADAMTS13 could have a pathophysiological link to PAH through its potentially altered local expression in the dysfunctional pulmonary vasculature. Although hepatic stellate cells account for most circulating ADAMTS13,^31^ an altered ADAMTS13 expression in the pulmonary circulation could entail local variations of plasma ADAMTS13 levels that we were not able to detect using a systemic venous blood test. This could then potentially be a contributor to the microvascular thrombosis found in PAH.^2^^,^^3^ Due to the previously mentioned limited amount of research of ADAMTS13 in PAH, it is therefore paramount for further studies examining and verifying this relationship and its possible causes.

### Strengths and Limitations

The use of PEA and its great sensitivity and specificity for measuring protein plasma levels is a strength of this study, despite not resulting in absolute concentrations but rather arbitrary units, because this was not needed for the objective of the study. However, this limits the possibility of using specific protein concentration cutoffs to compare protein levels with other studies, or with clinically used cutoffs or end points in other conditions. The use of RHC to obtain hemodynamic measurements and the patient cohort consisting of patients diagnosed by cardiologists experienced in PH are also considered strengths. Limitations include possible confounding factors such as medication intake and comorbidities in the patient groups. Although this study demonstrates associations between plasma ADAMTS13 and PAH, it does not examine any potential mechanistical explanations for this relationship, which can currently only be speculated on based on present knowledge on ADAMTS13 expression and regulation. Nonetheless, this study illuminates ADAMTS13 as a protein of interest in the diagnosis of PAH and serves as a basis for further investigation.

## Interpretation

We have previously demonstrated that plasma ADAMTS13 is a promising potential biomarker able to differentiate PAH from other PH subtypes including CTEPH, HFpEF-PH, and HFrEF-PH and a dyspnea HF-Non-PH control group. In this study with an expanded cohort and external validation, we confirm the ability of plasma ADAMTS13 to differentiate these groups independently of age and sex, thus indicating it as a possible biomarker candidate in PAH and highlighting the need for further independent studies in larger cohorts using multimarker panels.

## Funding/Support

The work was supported by unrestricted research grants from Avtal om Läkarutbildning och Forskning (ALF), Skåne University Hospital foundations and donations, and Go Rad Care AB. The work was also supported by the 10.13039/501100009708Novo Nordic Foundation [Grant NNF20OC0064556 to J. C.].

## Financial/Nonfinancial Disclosures

The authors have reported to *CHEST Pulmonary* the following: A. A. and S. A. report personal lecture fees from Janssen and Nordic Infucare, outside the submitted work. J. C. reports an unrestricted research grant from Actelion and institutional teaching fees outside the submitted work from Astra Zeneca, United Therapeutics, Nordic InfuCare, and Ferrer; and is or has been primary investigator or coinvestigator on clinical PAH trials for Acceleron, Actelion, GlaxoSmithKline, Janssen, MSD, Pfizer, and United Therapeutics. G. R. reports unrestricted research grants from ALF, Go Rad Care AB, and Skåne University Hospital during the conduct of the study; reports personal lecture fees from Actelion Pharmaceuticals Sweden AB, AOP Health/Orpha Care, Bayer HealthCare, Bohringer Ingelheim, GlaxoSmithKline, Janssen, MSD, and Nordic Infucare, outside the submitted work; and is or has been a primary investigator or coinvestigator in clinical PAH trials for Acceleron, Actelion Pharmaceuticals Sweden AB, Acceleron, Bayer HealthCare, GlaxoSmithKline, Janssen, MSD, Pfizer, and United Therapeutics and in clinical heart transplantation immunosuppression trials for Novartis. None declared (K. K., A. E. S., K. T.-L.).

## Supplementary Data

e-Online Data
