# Supplementary material for: Can Plasma ADAMTS13 Differentiate Patients With Pulmonary Arterial Hypertension From Other Forms of Pulmonary Hypertension and Dyspnea Control Patients
Source: CHEST Pulm. 2025 May 19;3(3):100178. doi: 10.1016/j.chpulm.2025.100178 (PMC13418282; doi:10.1016/j.chpulm.2025.100178)
Supplement: e-Online Data [file mmc1.docx]

**Supplementary figures and tables**

**e-Table 1.** The list of all included proteins, their abbreviations and their ID on www.UniProt.org

| **Abbreviation** | **Name** | **UniProt ID** |
| --- | --- | --- |
| 5'-NT | 5'-nucleotidase | P21589 |
| ADAMTS13 | A disintegrin and metalloproteinase with thrombospondin motifs 13 | Q76LX8 |
| AGRP | Agouti-related protein | O00253 |
| AMBP | Protein AMBP | P02760 |
| AP-N | Aminopeptidase N | P15144 |
| BMP-6 | Bone morphogenetic protein 6 | P22004 |
| BNP | Natriuretic peptides B | P16860 |
| BOC | Brother of CDO | Q9BWV1 |
| CA5A | Carbonic anhydrase 5A, mitochondrial | P35218 |
| CPA1 | Carboxypeptidase A1 | P15085 |
| CPB1 | Carboxypeptidase B | P15086 |
| CPE | Carboxypeptidase E | P16870 |
| CTRC | Chymotrypsin C | Q99895 |
| Dkk-1 | Dickkopf-related protein 1 | O94907 |
| DLK-1 | Protein delta homolog 1 | P80370 |
| DLL1 | Delta-like protein 1 | O00548 |
| FS | Follistatin | P19883 |
| FURIN | Furin | P09958 |
| GDF-2 | Growth/differentiation factor 2 | Q9UK05 |
| GH | Growth hormone | P01241 |
| - | Glyoxalase I | Q04760 |
| GT | Gastrotropin | P51161 |
| HAOX1 | Hydroxyacid oxidase 1 | Q9UJM8 |
| HO-1 | Heme oxygenase 1 | P09601 |
| HSP 27 | Heat shock 27 kDa protein | P04792 |
| IDUA | Alpha-L-iduronidase | P35475 |
| IF | Intrinsic factor | P27352 |
| MAD homolog 5 | Mothers against decapentaplegic homolog 5 | Q99717 |
| NEMO | NF-kappa-B essential modulator | Q9Y6K9 |
| Notch 3 | Neurogenic locus notch homolog protein 3 | Q9UM47 |
| PAI-1 | Plasminogen activator inhibitor 1 | P05121 |
| PAR-1 | Proteinase-activated receptor 1 | P25116 |
| PARP-1 | Poly [ADP-ribose] polymerase 1 | P09874 |
| PD-L2 | Programmed cell death 1 ligand 2 | Q9BQ51 |
| PODXL | Podocalyxin | O00592 |
| PPY | Pancreatic prohormone | P01298 |
| PRSS27 | Serine protease 27 | Q9BQR3 |
| PRSS8 | Prostasin | Q16651 |
| RSPO3 | R-spondin-3 | Q9BXY4 |
| SCAMP3 | Secretory carrier-associated membrane protein 3 | O14828 |
| SCGB3A2 | Secretoglobin family 3A member 2 | Q96PL1 |
| SEZ6L | Seizure 6-like protein | Q9BYH1 |
| SHPS-1 | Tyrosine-protein phosphatase non-receptor type substrate 1 | P78324 |
| SOD2 | Superoxide dismutase [Mn], mitochondrial | P04179 |
| SORT1 | Sortilin | Q99523 |
| SPON1 | Spondin-1 | Q9HCB6 |
| SPON2 | Spondin-2 | Q9BUD6 |
| STK4 | Serine/threonine-protein kinase 4 | Q13043 |
| TF | Tissue factor | P13726 |
| TFPI | Tissue factor pathway inhibitor | P10646 |
| TFPI-2 | Tissue factor pathway inhibitor 2 | P48307 |
| TGFR-2 | TGF-beta receptor type-2 | P37173 |
| TGM2 | Protein-glutamine gamma-glutamyltransferase 2 | P21980 |
| THPO | Thrombopoietin | P40225 |
| TM | Thrombomodulin | P07204 |
| t-PA | Tissue-type plasminogen activator | P00750 |
| uPA | Urokinase-type plasminogen activator | P00749 |
| uPAR | Urokinase plasminogen activator surface receptor | Q03405 |
| VSIG2 | V-set and immunoglobulin domain-containing protein 2 | Q96IQ7 |
| vWF | von Willebrand factor | P04275 |
| WIF-1 | Wnt inhibitory factor 1 | Q9Y5W5 |
| XPNPEP2 | Xaa-Pro aminopeptidase 2 | O43895 |

**e-Table 2.** Protein levels in the Lund discovery cohort

| **Protein (AU)** | **Controls (n = 55)** | **CTEPH (n = 54)** | **PAH (n = 95)** | **HFpEF-PH (n = 58)** | **HFrEF-PH (n = 64)** | **HF-Non-PH (n = 45)** |
| --- | --- | --- | --- | --- | --- | --- |
|  | **Median (IQR)** | **Median (IQR)** | **Median (IQR)** | **Median (IQR)** | **Median (IQR)** | **Median (IQR)** |
| **5'-NT** | 645 (517 - 726)a | 884 (654 - 1264) | 1022 (721 - 1505)c | 1000 (723 - 1666) | 1397 (885 - 1873)a | 858 (636 - 1389) |
| **ADAMTS13** | 25.6 (24.3 - 27.7) | 26.8 (24.5 - 29.6) | 24.8 (23.5 - 27.4) | 27.2 (24.1 - 30.5) | 27.3 (24.8 - 30.2) | 25.5 (24.2 - 28.3) |
| **AGRP** | 6.34 (5.19 - 8.04) | 6.15 (4.79 - 7.52) | 6.81 (5.15 - 9.89) | 6.55 (5.39 - 8.74) | 7.36 (6 - 9.79) | 5.84 (4.76 - 7.82) |
| **AMBP** | 88.7 (81.5 - 99.1) | 93.8 (84.6 - 108) | 93.7 (83.4 - 104) | 105 (90.2 - 120) | 91.4 (78 - 103) | 93.3 (82.7 - 109) |
| **AP-N** | 21.5 (19.2 - 24.3) | 22.5 (18.7 - 27) | 22.4 (18.6 - 28.3) | 23.7 (19.6 - 32.2) | 27.7 (22.4 - 37) | 22.3 (18.9 - 27.2) |
| **BMP-6** | 28 (24.1 - 32.8) | 36 (28.9 - 42.9) | 35.9 (28.1 - 45.4) | 37.4 (32.1 - 48.3) | 46.3 (35.9 - 60.4) | 34.2 (27.9 - 45.1) |
| **BNP** | 2.17 (1.44 - 3.25) | 31.8 (5.59 - 78.2) | 41.2 (11.2 - 96.8) | 27.9 (16.3 - 49.3) | 143 (63.8 - 186) | 28.5 (5.32 - 75) |
| **BOC** | 24.6 (22.5 - 28.2) | 21.3 (18.8 - 25.8) | 23.1 (19.9 - 28.1) | 23.5 (20.3 - 27.1) | 25.2 (22 - 32.1) | 23.5 (20.7 - 26.3) |
| **CA5A** | 1.9 (1.37 - 2.8) | 2.97 (2.05 - 4.39) | 3.03 (2.07 - 5.63) | 4.98 (2.81 - 8.03) | 5.66 (3.3 - 10.4) | 3.66 (2.15 - 6.62) |
| **CPA1** | 18.9 (12.6 - 23.3) | 21.2 (13 - 35.1) | 20 (14.5 - 27.2) | 23.2 (17.4 - 39.1) | 26.8 (18.7 - 39.4) | 20.2 (16.6 - 28.1) |
| **CPB1** | 12.1 (8.8 - 15.5) | 13.6 (9.26 - 19.9) | 14.1 (9.27 - 17.4) | 15.9 (10.7 - 20.4) | 17.7 (13.3 - 25.2) | 13.1 (9.78 - 20.7) |
| **CPE** | 9.57 (8.16 - 11.5)a | 10.5 (8.85 - 12.4) | 10.4 (8.78 - 11.5)c | 9.87 (8.47 - 11.6) | 11.6 (9.99 - 13.3)a | 10.6 (9.02 - 11.9) |
| **CTRC** | 1269 (950 - 1852) | 1292 (815 - 1568) | 1146 (807 - 1425) | 1162 (825 - 1656) | 1324 (875 - 1788) | 1102 (730 - 1719) |
| **Dkk-1** | 222 (157 - 317) | 245 (186 - 340) | 269 (191 - 380) | 291 (215 - 409) | 241 (181 - 334) | 240 (187 - 380) |
| **DLK-1** | 16.3 (10.7 - 24.4) | 13.6 (9.46 - 21.5) | 14.7 (11 - 23.1) | 18.8 (13.4 - 32.2) | 17.5 (12.3 - 27.3) | 14.8 (11 - 23.1) |
| **DLL1** | 340 (283 - 389)a | 356 (278 - 473) | 392 (312 - 506)c | 495 (374 - 619) | 433 (353 - 594)a | 382 (307 - 483) |
| **FS** | 1904 (1420 - 2473) | 2490 (2033 - 3013) | 2451 (1988 - 3189) | 2692 (2070 - 3469) | 2722 (2055 - 3477) | 2474 (1809 - 3024) |
| **FURIN** | 9.18 (7.59 - 11.1)a | 10.9 (9.12 - 12.7) | 10.5 (9.05 - 12.4)c | 10.6 (8.76 - 13.1) | 9.98 (8.68 - 11.8)a | 10.6 (8.41 - 12.3) |
| **GDF-2** | 22.2 (16.4 - 27.8) | 16.2 (13 - 21.5) | 18.9 (14.6 - 25.1) | 16.1 (11.6 - 21.1) | 20 (14.4 - 23.7) | 17.4 (13.9 - 23.1) |
| **GH** | 56.3 (22.4 - 177) | 163 (49.3 - 550) | 218 (108 - 735) | 238 (113 - 568) | 469 (80.3 - 1549) | 92.9 (28.9 - 644) |
| **IF** | 39.6 (28.7 - 59.7) | 41.6 (27 - 90.2) | 42.6 (30.8 - 73.2) | 50.5 (29.6 - 87.3) | 34.5 (23.6 - 53.1) | 56.6 (31.4 - 77.7) |
| **Glyoxalase I** | 57.8 (37.4 - 82.6) | 114 (65.9 - 249) | 86.9 (54.3 - 140) | 132 (63.8 - 246) | 161 (80.5 - 266) | 90.7 (49.5 - 165) |
| **GT** | 2.06 (1.6 - 2.62) | 2.9 (2.3 - 3.84) | 2.42 (1.83 - 3.25) | 2.66 (2.11 - 3.82) | 2.68 (2.14 - 3.88) | 2.67 (2.01 - 4.24) |
| **HAOX1** | 8.23 (5.11 - 18.1) | 13.8 (9.38 - 22.4) | 18.6 (8.66 - 41.1) | 18.5 (9.85 - 44.1) | 25.9 (12.2 - 69.8) | 16.8 (7.21 - 38.5) |
| **HO-1** | 1296 (1164 - 1534) | 1583 (1305 - 1922) | 1414 (1172 - 1801) | 1588 (1236 - 1949) | 1513 (1321 - 1920) | 1396 (1141 - 1901) |
| **HSP 27** | 1544 (1358 - 1738) | 1515 (1324 - 1674) | 1468 (1343 - 1665) | 1566 (1384 - 1686) | 1463 (1276 - 1605) | 1564 (1431 - 1719) |
| **IDUA** | 21.6 (18.7 - 26.8) | 24.7 (19.3 - 30.6) | 24 (20.2 - 27.9) | 25.1 (21 - 32.6) | 26.8 (21.3 - 32.8) | 22.5 (16.9 - 29.6) |
| **MAD homolog 5** | 8.29 (7.92 - 8.72)a | 8.57 (8.3 - 9.1) | 8.51 (7.98 - 8.96)c | 8.84 (8.25 - 9.38) | 8.66 (8.11 - 9.38)a | 8.62 (8.34 - 8.92) |
| **NEMO** | 76.5 (35.1 - 132) | 90.7 (55.1 - 145) | 86.2 (53.9 - 177) | 110 (65.9 - 207) | 101 (58.1 - 216) | 93 (60.6 - 179) |
| **Notch 3** | 7.79 (6.79 - 9.48) | 11 (8.01 - 13.5) | 10.6 (8.84 - 14.3) | 12.8 (10.4 - 15.1) | 11.5 (9.56 - 15.7) | 9.6 (8.67 - 12.8) |
| **PAI** | 32.5 (20.8 - 51.1) | 47.2 (31.9 - 84.9) | 44.8 (30 - 80) | 48.3 (34.2 - 72.5) | 52.3 (30.6 - 86.7) | 47.3 (34.5 - 80.2) |
| **PAR-1** | 140 (117 - 183) | 155 (130 - 202) | 157 (130 - 189) | 199 (153 - 230) | 180 (134 - 215) | 161 (127 - 202) |
| **PARP-1** | 3.8 (3.29 - 4.69) | 4.69 (3.73 - 5.62) | 4.83 (4.03 - 6.28) | 4.1 (3.45 - 5.06) | 5.16 (3.77 - 6.53) | 4.53 (3.55 - 5.43) |
| **PD-L2** | 4.65 (4.07 - 5.31) | 5.3 (4.27 - 6.73) | 5.71 (4.76 - 6.66) | 6.33 (5.24 - 7.44) | 6.17 (5.22 - 8.05) | 5.66 (4.47 - 6.66) |
| **PODXL** | 10.4 (9.51 - 11.1)a | 9.95 (9.23 - 10.9) | 10.1 (8.73 - 11.1)c | 9.88 (9.01 - 10.9) | 9.58 (8.13 - 10.3)a | 9.81 (9.04 - 10.6) |
| **PPY** | 49.3 (22.6 - 121)a | 62.2 (35.5 - 110) | 45.2 (26.8 - 89.3)c | 73.6 (29.3 - 130) | 82.3 (30.6 - 132)a | 61.3 (17.5 - 147) |
| **PRSS27** | 209 (181 - 268) | 180 (149 - 225) | 196 (157 - 252) | 210 (168 - 254) | 182 (146 - 241) | 205 (163 - 271) |
| **PRSS8** | 308 (263 - 421) | 433 (358 - 563) | 461 (350 - 553)a | 449 (363 - 514) | 415 (365 - 514) | 419 (327 - 543) |
| **RSPO3** | 7.29 (5.86 - 9.1)a | 12.2 (8.76 - 16.5) | 13.1 (10.1 - 16.8)c | 16 (11.3 - 22.2) | 15 (9.94 - 18.9)a | 11.6 (8.65 - 15.7) |
| **SCAMP3** | 24.9 (13.7 - 48.8)a | 37 (21.5 - 49.7) | 37.5 (19.5 - 62.4)c | 41.9 (27.5 - 63.6) | 30 (18 - 60.7)a | 43 (26.3 - 66.4) |
| **SCGB3A2** | 3.17 (2.6 - 3.98) | 4.12 (2.52 - 5.4) | 4.45 (2.62 - 8.58) | 4.91 (3.16 - 8.3) | 4.34 (3.16 - 7.52) | 3.3 (2.35 - 5.59) |
| **SEZ6L** | 24.4 (20.8 - 27.3)a | 23.8 (21.7 - 27.1) | 23.4 (20.4 - 27.4)c | 24.2 (18.9 - 29.7) | 24.2 (20.3 - 30)a | 24.1 (21.5 - 28.9) |
| **SHPS-1** | 6.92 (5.87 - 8.78) | 7.44 (6.24 - 9.63) | 7.28 (5.98 - 9.13) | 9.55 (7.37 - 12.1) | 9.46 (6.87 - 12.6) | 7.47 (6.01 - 10.4) |
| **SOD2** | 296 (284 - 323) | 291 (273 - 307) | 302 (280 - 333) | 298 (290 - 328) | 316 (293 - 367) | 299 (282 - 318) |
| **SORT1** | 52.9 (43 - 62) | 61.2 (54.6 - 71.3) | 61.5 (55.3 - 72.6) | 65 (58.4 - 72.5) | 64.4 (58.3 - 72.7) | 60.3 (48.8 - 71.8) |
| **SPON1** | 2.92 (2.58 - 3.25) | 3.37 (2.88 - 3.92) | 3.67 (3.11 - 4.27) | 4.09 (3.33 - 4.61) | 4.14 (3.41 - 5.11) | 3.41 (3.04 - 4.09) |
| **SPON2** | 283 (262 - 310) | 359 (325 - 400) | 383 (333 - 410) | 393 (357 - 430) | 394 (355 - 435) | 362 (333 - 397) |
| **STK4** | 17 (10.5 - 26) | 19.2 (13 - 24.2) | 19.5 (12.4 - 28.8) | 23.1 (13.3 - 29.5) | 19.5 (11.3 - 29) | 20.8 (14.3 - 28.6) |
| **TF** | 31.6 (28.3 - 35.9) | 41.2 (30.8 - 48.3) | 36.3 (30.6 - 42.5) | 45.8 (36.1 - 60.6) | 40 (32.7 - 49.2) | 37.1 (31.1 - 47.4) |
| **TFPI** | 235 (201 - 275) | 215 (189 - 249) | 223 (180 - 261) | 238 (194 - 297) | 262 (225 - 308) | 221 (195 - 256) |
| **TFPI-2** | 133 (108 - 172)a | 190 (159 - 257) | 242 (184 - 299)c | 217 (173 - 283) | 235 (201 - 383)a | 206 (160 - 246) |
| **TGFR-2** | 70.8 (60.8 - 88.7)a | 90.4 (60.2 - 110) | 90.5 (67.3 - 125)c | 127 (87.6 - 169) | 102 (75.8 - 139)a | 95.7 (74.8 - 119) |
| **TGM2** | 200 (143 - 252) | 236 (195 - 284) | 247 (188 - 309) | 194 (148 - 231) | 219 (156 - 270) | 214 (156 - 275) |
| **THPO** | 4.25 (3.68 - 5.08) | 4.24 (3.67 - 4.94) | 4.2 (3.51 - 5.01) | 4.8 (3.88 - 5.42) | 4.13 (3.45 - 4.83) | 3.91 (3.49 - 4.91) |
| **TM** | 242 (205 - 302) | 239 (189 - 287) | 235 (188 - 294) | 326 (235 - 424) | 272 (208 - 345) | 261 (199 - 320) |
| **t-PA** | 21.8 (14.5 - 30.7) | 40.7 (24.9 - 58.4) | 38.9 (26.4 - 61.5) | 39.5 (29.2 - 65.5) | 40.7 (29.8 - 59.5) | 36.3 (25.2 - 53) |
| **uPA** | 19.4 (15.4 - 21.6) | 18.1 (15 - 20.9) | 18.3 (15.1 - 23.3) | 19.8 (16.8 - 23.6) | 20.7 (17.2 - 27) | 18.7 (15.8 - 20.8) |
| **U-PAR** | 9.93 (8.55 - 11.8) | 15.8 (12.3 - 20.7) | 17 (12.6 - 21.6) | 20.3 (13.7 - 26.4) | 17.5 (14.2 - 26.6) | 14.7 (11.1 - 20.1) |
| **VSIG2** | 6.69 (5.4 - 8.58) | 9.9 (6.85 - 14.3) | 10.5 (6.9 - 16.7) | 14.6 (8.02 - 22.1) | 13.1 (8.47 - 17.4) | 9.1 (6.09 - 19.7) |
| **vWF** | 51.5 (33.7 - 78.4) | 70.3 (43.9 - 134) | 79.4 (45.2 - 149) | 82.9 (57.7 - 151) | 92.5 (54.1 - 134) | 68.9 (45.8 - 112) |
| **WIF-1** | 39.6 (32.7 - 45.8)a | 44.6 (35.8 - 56.3) | 46.4 (36.9 - 58.8)c | 43 (33.2 - 54.3) | 57.9 (47.6 - 71.6)a | 45.5 (34.1 - 56.9) |
| **XPNPEP2** | 83.4 (58.1 - 113)a | 98.1 (53.1 - 122) | 78.4 (49.3 - 110)c | 71.3 (38.6 - 102) | 89.6 (47.6 - 124)a | 76.9 (44.1 - 107) |

AU indicates arbitrary units; CTEPH, chronic thromboembolic pulmonary hypertension; HF-Non-PH, heart failure without pulmonary hypertension; HFpEF-PH, heart failure with preserved ejection fraction with pulmonary hypertension; HFrEF-PH, heart failure with reserved ejection fraction with pulmonary hypertension; IQR, interquartile range and PAH, pulmonary arterial hypertension;

a = n-1.

b = n-2

c = n-3

**e-Table 3.** Results from the Kruskal-Wallis test of all 61 proteins to find if the levels of PAH patients in the discovery cohort differs against any of the 4 other patient groups

| **Protein** | ***p* value** | **Protein** | ***p* value** |
| --- | --- | --- | --- |
| 5'-NT | 0.001* | PAR-1 | 0.003* |
| ADAMTS13 | 0.001* | PARP-1 | 0.005* |
| AGRP | 0.011 | PD-L2 | 0.008 |
| AMBP | 0.010 | PODXL | 0.219 |
| AP-N | 0.001* | PPY | 0.195 |
| BMP-6 | 0.001* | PRSS27 | 0.219 |
| BOC | 0.007 | PRSS8 | 0.818 |
| CA5A | <0.001* | RSPO3 | 0.008 |
| CPA1 | 0.012 | SCAMP3 | 0.276 |
| CPB1 | 0.006 | SCGB3A2 | 0.039 |
| CPE | 0.004* | SEZ6L | 0.939 |
| CTRC | 0.540 | SHPS-1 | <0.001* |
| Dkk-1 | 0.184 | SOD2 | <0.001* |
| DLK-1 | 0.007 | SORT1 | 0.100 |
| DLL1 | <0.001* | SPON1 | <0.001* |
| FS | 0.266 | SPON2 | <0.001* |
| FURIN | 0.430 | STK4 | 0.382 |
| GDF-2 | 0.034 | TF | 0.001* |
| GH | 0.004* | TFPI | <0.001* |
| IF | 0.023 | TFPI-2 | 0.005 |
| Glyoxalase I | 0.002* | TGFR-2 | <0.001* |
| GT | 0.160 | TGM2 | <0.001* |
| HAOX1 | 0.046 | THPO | 0.134 |
| HO-1 | 0.155 | TM | <0.001* |
| HSP 27 | 0.047 | t-PA | 0.654 |
| IDUA | 0.120 | uPA | 0.011 |
| MAD homolog 5 | 0.047 | U-PAR | 0.008 |
| NEMO | 0.394 | VSIG2 | 0.070 |
| Notch 3 | 0.002* | vWF | 0.253 |
| PAI | 0.932 | WIF-1 | <0.001* |
|  |  | XPNPEP2 | 0.097 |

**p* < 0.005

**e-Table 4.** Comparisons of protein levels between groups in the Lund discovery cohort.

| **Protein** | **Comparison** | ***p* value** |  | **Protein** | **Comparison** | ***p* value** |
| --- | --- | --- | --- | --- | --- | --- |
| **5'-NT** | PAH vs. CTEPH | 0.064 |  | **PARP-1** | PAH vs. CTEPH | 0.263 |
|  | PAH vs. HFpEF-PH | 0.994 |  |  | PAH vs. HFpEF-PH | 0.002* |
|  | PAH vs. HFrEF-PH | 0.015* |  |  | PAH vs. HFrEF-PH | 0.598 |
|  | PAH vs. HF-Non-PH | 0.168 |  |  | PAH vs. HF-Non-PH | 0.090 |
| **ADAMTS13** | PAH vs. CTEPH | 0.004* |  | **SHPS-1** | PAH vs. CTEPH | 0.736 |
|  | PAH vs. HFpEF-PH | <0.001* |  |  | PAH vs. HFpEF-PH | <0.001* |
|  | PAH vs. HFrEF-PH | <0.001* |  |  | PAH vs. HFrEF-PH | <0.001* |
|  | PAH vs. HF-Non-PH | 0.043* |  |  | PAH vs. HF-Non-PH | 0.389 |
| **AP-N** | PAH vs. CTEPH | 0.980 |  | **SOD2** | PAH vs. CTEPH | 0.027* |
|  | PAH vs. HFpEF-PH | 0.188 |  |  | PAH vs. HFpEF-PH | 0.719 |
|  | PAH vs. HFrEF-PH | <0.001* |  |  | PAH vs. HFrEF-PH | 0.007* |
|  | PAH vs. HF-Non-PH | 0.786 |  |  | PAH vs. HF-Non-PH | 0.620 |
| **BMP-6** | PAH vs. CTEPH | 0.465 |  | **SPON1** | PAH vs. CTEPH | 0.102 |
|  | PAH vs. HFpEF-PH | 0.403 |  |  | PAH vs. HFpEF-PH | 0.020* |
|  | PAH vs. HFrEF-PH | <0.001* |  |  | PAH vs. HFrEF-PH | 0.004* |
|  | PAH vs. HF-Non-PH | 0.595 |  |  | PAH vs. HF-Non-PH | 0.541 |
| **CA5A** | PAH vs. CTEPH | 0.579 |  | **SPON2** | PAH vs. CTEPH | 0.110 |
|  | PAH vs. HFpEF-PH | 0.002* |  |  | PAH vs. HFpEF-PH | 0.028* |
|  | PAH vs. HFrEF-PH | <0.001* |  |  | PAH vs. HFrEF-PH | 0.014* |
|  | PAH vs. HF-Non-PH | 0.377 |  |  | PAH vs. HF-Non-PH | 0.315 |
| **CPE** | PAH vs. CTEPH | 0.425 |  | **TF** | PAH vs. CTEPH | 0.140 |
|  | PAH vs. HFpEF-PH | 0.301 |  |  | PAH vs. HFpEF-PH | <0.001* |
|  | PAH vs. HFrEF-PH | 0.002* |  |  | PAH vs. HFrEF-PH | 0.044* |
|  | PAH vs. HF-Non-PH | 0.631 |  |  | PAH vs. HF-Non-PH | 0.217 |
| **DLL1** | PAH vs. CTEPH | 0.103 |  | **TFPI** | PAH vs. CTEPH | 0.579 |
|  | PAH vs. HFpEF-PH | 0.002* |  |  | PAH vs. HFpEF-PH | 0.061 |
|  | PAH vs. HFrEF-PH | 0.047* |  |  | PAH vs. HFrEF-PH | <0.001* |
|  | PAH vs. HF-Non-PH | 0.911 |  |  | PAH vs. HF-Non-PH | 0.821 |
| **GH** | PAH vs. CTEPH | 0.154 |  | **TGFR-2** | PAH vs. CTEPH | 0.328 |
|  | PAH vs. HFpEF-PH | 0.825 |  |  | PAH vs. HFpEF-PH | <0.001* |
|  | PAH vs. HFrEF-PH | 0.083 |  |  | PAH vs. HFrEF-PH | 0.177 |
|  | PAH vs. HF-Non-PH | 0.027* |  |  | PAH vs. HF-Non-PH | 0.759 |
| **Glyoxalase I** | PAH vs. CTEPH | 0.012* |  | **TGM2** | PAH vs. CTEPH | 0.485 |
|  | PAH vs. HFpEF-PH | 0.011* |  |  | PAH vs. HFpEF-PH | <0.001* |
|  | PAH vs. HFrEF-PH | <0.001* |  |  | PAH vs. HFrEF-PH | 0.020* |
|  | PAH vs. HF-Non-PH | 0.491 |  |  | PAH vs. HF-Non-PH | 0.029* |
| **Notch 3** | PAH vs. CTEPH | 0.838 |  | **TM** | PAH vs. CTEPH | 0.767 |
|  | PAH vs. HFpEF-PH | 0.006* |  |  | PAH vs. HFpEF-PH | <0.001* |
|  | PAH vs. HFrEF-PH | 0.034* |  |  | PAH vs. HFrEF-PH | 0.035* |
|  | PAH vs. HF-Non-PH | 0.280 |  |  | PAH vs. HF-Non-PH | 0.215 |
| **PAR-1** | PAH vs. CTEPH | 0.990 |  | **WIF-1** | PAH vs. CTEPH | 0.569 |
|  | PAH vs. HFpEF-PH | <0.001* |  |  | PAH vs. HFpEF-PH | 0.212 |
|  | PAH vs. HFrEF-PH | 0.042* |  |  | PAH vs. HFrEF-PH | <0.001* |
|  | PAH vs. HF-Non-PH | 0.470 |  |  | PAH vs. HF-Non-PH | 0.403 |

CTEPH indicates chronic thromboembolic pulmonary hypertension; HF-Non-PH, heart failure without pulmonary hypertension; HFpEF-PH, heart failure with preserved ejection fraction with pulmonary hypertension; HFrEF-PH, heart failure with reserved ejection fraction with pulmonary hypertension;

**p* < 0.05

**e-Table 5.** Protein levels in the Lund discovery cohort PAH population compared to healthy controls

| **Protein** | ***p* value** |  | **Protein** | ***p* value** |
| --- | --- | --- | --- | --- |
| 5'-NT | <0.001* |  | PAR-1 | 0.092 |
| ADAMTS13 | 0.087 |  | PARP-1 | <0.001* |
| AGRP | 0.256 |  | PD-L2 | <0.001* |
| AMBP | 0.172 |  | PODXL | 0.172 |
| AP-N | 0.344 |  | PPY | 0.835 |
| BMP-6 | <0.001* |  | PRSS27 | 0.149 |
| BOC | 0.028 |  | PRSS8 | <0.001* |
| CA5A | <0.001* |  | RSPO3 | <0.001* |
| CPA1 | 0.321 |  | SCAMP3 | 0.021 |
| CPB1 | 0.195 |  | SCGB3A2 | 0.001* |
| CPE | 0.137 |  | SEZ6L | 0.646 |
| CTRC | 0.044 |  | SHPS-1 | 0.276 |
| Dkk-1 | 0.050 |  | SOD2 | 0.600 |
| DLK-1 | 0.619 |  | SORT1 | <0.001* |
| DLL1 | 0.002* |  | SPON1 | <0.001* |
| FS | <0.001* |  | SPON2 | <0.001* |
| FURIN | 0.002* |  | STK4 | 0.416 |
| GDF-2 | 0.026 |  | TF | 0.003* |
| GH | <0.001* |  | TFPI | 0.115 |
| IF | 0.401 |  | TFPI-2 | <0.001* |
| Glyoxalase I | <0.001* |  | TGFR-2 | 0.002* |
| GT | 0.020 |  | TGM2 | 0.001* |
| HAOX1 | <0.001* |  | THPO | 0.744 |
| HO-1 | 0.049 |  | TM | 0.377 |
| HSP 27 | 0.234 |  | t-PA | <0.001* |
| IDUA | 0.082 |  | uPA | 0.975 |
| MAD homolog 5 | 0.150 |  | U-PAR | <0.001* |
| NEMO | 0.082 |  | VSIG2 | <0.001* |
| Notch 3 | <0.001* |  | vWF | <0.001* |
| PAI | 0.005* |  | WIF-1 | 0.004* |
|  |  |  | XPNPEP2 | 0.427 |

**p* < 0.008
